# Bacterial pathogens in Xpert MTB/RIF Ultra-negative sputum samples of patients with presumptive tuberculosis in a high TB burden setting: a 16S rRNA analysis

**DOI:** 10.1128/spectrum.02931-23

**Published:** 2024-01-08

**Authors:** Wakjira Kebede, Gemeda Abebe, Ilke De Boeck, Esayas Kebede Gudina, Eline Cauwenberghs, Sarah Lebeer, Annelies Van Rie

**Affiliations:** 1 Department of Epidemiology and Social Medicine, Faculty of Medicine and Health sciences, University of Antwerp, Antwerp, Belgium; 2 Mycobacteriology Research Center, Jimma University, Jimma, Ethiopia; 3 School of Medical Laboratory Science, Jimma University, Jimma, Ethiopia; 4 Department of Bioscience Engineering, University of Antwerp, Antwerp, Belgium; 5 Department of Internal Medicine, Jimma University Medical Center, Jimma University, Jimma, Ethiopia; University of Manitoba, Winnipeg, Manitoba, Canada

**Keywords:** diagnostics, presumptive TB cases, *M. tuberculosis*, bacterial etiology, LRTIs, sequencing, Ethiopia

## Abstract

In patients with presumptive tuberculosis (TB) in whom the diagnosis of TB was excluded, understanding the bacterial etiology of lower respiratory tract infections (LRTIs) is important for optimal patient management. A secondary analysis was performed on a cohort of 250 hospitalized patients with symptoms of TB. Bacterial DNA was extracted from sputum samples for Illumina 16S rRNA sequencing to identify bacterial species based on amplicon sequence variant level. The bacterial pathogen most likely to be responsible for the patients’ LRTI could only be identified in a minority (6.0%, 13/215) of cases based on 16S rRNA amplicon sequencing: *Mycoplasma pneumoniae* (*n* = 7), *Bordetella pertussis* (*n* = 2), *Acinetobacter baumanii* (*n* = 2), and *Pseudomonas aeruginosa* (*n* = 2). Other putative pathogens were present in similar proportions of Xpert Ultra-positive and Xpert Ultra-negative sputum samples. The presence of *Streptococcus* (*pseudo*)*pneumoniae* appeared to increase the odds of radiological abnormalities (aOR 2.5, 95% CI 1.12–6.16) and the presence of *S.* (*pseudo*)*pneumoniae* (aOR 5.31, 95% CI 1.29–26.6) and *Moraxella catarrhalis/nonliquefaciens* (aOR 12.1, 95% CI 2.67–72.8) increased the odds of 6-month mortality, suggesting that these pathogens might have clinical relevance. *M. pneumoniae*, *B. pertussis,* and *A. baumanii* appeared to be the possible causes of TB-like symptoms. *S.* (*pseudo*)*pneumoniae* and *M. catarrhalis*/*nonliquefaciens* also appeared of clinical relevance based on 16S rRNA amplicon sequencing. Further research using tools with higher discriminatory power than 16S rRNA sequencing is required to develop optimal diagnostic and treatment strategies for this population.

**IMPORTANCE:**

The objective of this study was to identify possible bacterial lower respiratory tract infection (LRTI) pathogens in hospitalized patients who were initially suspected to have TB but later tested negative using the Xpert Ultra test. Although 16S rRNA was able to identify some less common or difficult-to-culture pathogens such as *Mycoplasma pneumoniae* and *Bordetella pertussis*, one of the main findings of the study is that, in contrast to what we had hypothesized, 16S rRNA is not a method that can be used to assist in the management of patients with presumptive TB having a negative Xpert Ultra test. Even though this could be considered a negative finding, we believe it is an important finding to report as it highlights the need for further research using different approaches.

## INTRODUCTION

Lower respiratory tract infections (LRTIs), which include bronchitis, bronchiolitis, and pneumonia, are one of the most common diseases, with 489 million LRTI episodes occurring annually worldwide (1, 2). Globally, LRTIs are the fourth leading cause of death claiming 2.4 million lives in 2019 (2). In Ethiopia, LRTIs are the main reason for hospital admissions and the third leading cause of death, accounting for 8.2% of all deaths in 2019 (3).

LRTIs are caused by a range of pathogens, including bacteria, viruses, and fungi (3–5). The main bacterial etiologies of LTRIs are *Mycobacterium tuberculosis* (*Mtb*)*, Streptococcus pneumoniae, Haemophilus influenzae, Klebsiella pneumoniae, Staphylococcus aureus, Acinetobacter* species (spp.)*, Streptococcus viridans, Pseudomonas aeruginosa, Escherichia coli,* and *Proteus* spp. (4, 6–8). Atypical pathogens that can cause LRTIs are *Mycoplasma pneumoniae, Chlamydia pneumoniae,* and *Legionella pneumophila* (9).

In most low- and middle-income countries, people presenting with prolonged cough are first investigated for tuberculosis (TB) using smear microscopy or a rapid molecular test, such as the Xpert MB/RIF assay. When *Mtb* is not detected, clinicians often prescribe a trial of broad-spectrum antibiotics (10, 11). This can be problematic as empiric use of antibiotics does not always result in clinical improvement and may drive the emergence of antibiotic resistance (12). When broad-spectrum antibiotics fail to improve clinical symptoms, empiric TB treatment is often initiated even though studies have shown that empiric TB treatment does not affect survival (13–15) and may even increase mortality (16).

In 2017, the World Health Organization (WHO) endorsed the Xpert Ultra assay (17) given its excellent performance for the diagnosis of TB, with a pooled sensitivity of 88% (95% CI: 85% to 91%) and specificity of 96% (95% CI: 94% to 97%) compared to liquid culture (18, 19). With a high negative predictive value (98.1%), patients presenting with symptoms of TB whose sputum sample is Xpert Ultra negative are thus highly unlikely to suffer from pulmonary TB (20). This raises the question whether empiric TB treatment for patients with negative Xpert Ultra test results is the correct management. A better understanding of the etiological cause of respiratory symptoms in patients presenting with symptoms of TB whose sputum sample is negative on Xpert Ultra is important to develop evidence-based algorithms for the optimal management of this patient population.

16S rRNA gene amplicon sequencing is a culture-free method to identify and compare bacterial diversity and microbial composition of a sputum sample. 16S rRNA detects both culturable and non-culturable microorganisms (21) and is a less costly method for studying microbial diversity compared to whole genome sequencing and metagenomic approaches (22). When informative, implementation of a 16S RNA assay could help clinicians make decisions and implement effective therapeutic strategies, as has been done for patients with non-cystic fibrosis and chronic obstructive pulmonary disease (COPD) (23, 24). 16S rRNA gene amplicon sequencing has not yet been applied to study the prevalence of bacterial pathogens in sputum samples of patients presenting with symptoms of TB in whom the Xpert Ultra test result was negative.

This study aimed to use Illumina MiSeq 16S rRNA V4 amplicon sequencing to determine the putative etiology of LRTI in hospitalized presumptive TB patients in whom Mtb was not detected by the Xpert Ultra assay, as this information could result in the development of an assay to guide the management for this population. In addition, we aimed to compare the prevalence and distribution of respiratory bacterial pathogens in sputum samples that were Xpert Ultra positive and negative to assess whether differences in microbial composition observed by 16S rRNA could be a marker of the etiology of the respiratory symptoms. Finally, the association between the presence of specific bacterial pathogenic taxa in Xpert Ultra negative sputum sample and clinical improvement on an antibiotic trial, chest X-ray findings, and 6-month survival was explored to assess the clinical relevance of the bacterial composition of the sputum sample.

## RESULTS

### Cohort characteristics

Of the 250 Xpert MTB/RIF-negative participants presenting with symptoms suggestive of pulmonary TB, 35 (14%) were diagnosed with pulmonary TB (Xpert Ultra and culture positive) and 215 (86%) were not diagnosed with pulmonary TB (211 Xpert Ultra negative and culture negative; 4 Xpert Ultra negative and contaminated cultures). Among the 215 Xpert Ultra negative patients, 17.2% (*n* = 37) had a history of TB treatment, 20.2% (*n* = 42) were living with HIV, 13.5% (*n* = 29) were elderly (age ≥65 years), 6.3% (*n* = 13) were severely ill, 1.9% (*n* = 4) had diabetes mellitus (DM), and 5.1% (*n* = 11) had a diagnosis of COPD. Most patients had a normal chest X-ray (*n* = 150, 70.8%) and about half (*n* = 117, 54.4%) improved clinically after a trial of broad-spectrum antibiotics. Compared to participants with a positive Xpert Ultra test result, those with a negative Xpert Ultra test were less likely to have prolonged symptoms or comorbidity (diagnosis of DM or COPD) and were more likely to be older, underweight or overweight, have a normal chest X-ray and improve clinically after a trial of antibiotics (Table 1).

**TABLE 1:** Characteristics of 250 hospitalized adults with presumptive tuberculosis (TB) who tested negative on Xpert MTB/RIF, stratified by Xpert Ultra results

Characteristics	Category	Xpert Ultra negative N (%)	Xpert Ultra positive N (%)
All patients		215 (86.0)	35 (14.0)
Age	40 years	113 (52.6)	31 (88.6)
	41–64 years	73 (34.0)	2 (5.7)
	≥65 years	29 (13.5)	2 (5.7)
Sex	Female	117 (54.4)	21 (60.0)
	Male	98 (45.6)	14 (40.0)
Residence	Urban	92 (42.8)	14 (40.0)
	Rural	123 (57.2)	21 (60.0)
Body mass index	Underweight (<18.5 kg·m^−2^)	99 (46.0)	10 (28.6)
	Normal (18.5–24.9 kg·m^−2^)	61 (28.4)	23 (65.7)
	Overweight (>25–29.9 kg·m^−2^)	55 (25.6)	2 (5.7)
Co-morbidities	Diabetes mellitus	4 (1.9)	3 (8.6)
	Chronic obstructive pulmonary disease	11 (5.1)	7 (20.0)
HIV status^*a*^	HIV infected	42 (20.2)	10 (28.6)
	HIV negative—severely ill^*b*^	13 (6.3)	5 (14.3)
	HIV negative—not severely ill	153 (73.6)	20 (57.1)
History of TB treatment	No	178 (82.8)	27 (77.1)
	Yes	37 (17.2)	8 (22.9)
Clinical improvement on antibiotic trial	No	98 (44.6)	31 (88.6)
	Yes	117 (54.4)	4 (11.4)
Symptoms at presentation	Cough ≥2 weeks	134 (62.3)	31 (88.6)
	Shortness of breath ≥2 weeks	101 (47.0)	26 (74.3)
	Night sweat ≥2 weeks	102 (47.4)	18 (31.4)
	Fever ≥2 weeks	94 (43.7)	19 (54.3)
	Weight loss	112 (52.1)	27 (77.1)
	Loss of appetite	168 (78.1)	33 (94.3)
	Chest pain	137 (63.7)	31 (88.6)
Radiological findings	Normal	150 (70.8)	4 (11.4)
	Cavitary lesion	20 (9.4)	11 (31.4)
	Pleural effusion	20 (9.4)	6 (17.1)
	Consolidation	10 (4.7)	6 (17.1)
	Miliary disease	8 (3.8)	6 (17.1)
	Fibrosis	3 (1.4)	1 (2.9)
	Hilary adenopathy	1 (0.5)	1 (2.9)

^*a*^ HIV status missing for seven Xpert Ultra negative patients.

^*b*^ Severely ill-defined as temperature  >39°C, respiratory rate  > 30 resp./min, cardiac rate  >120 bpm, or unable to walk without help.

### Bacterial composition of sputum samples using 16S rRNA sequencing

**Fig 1:**
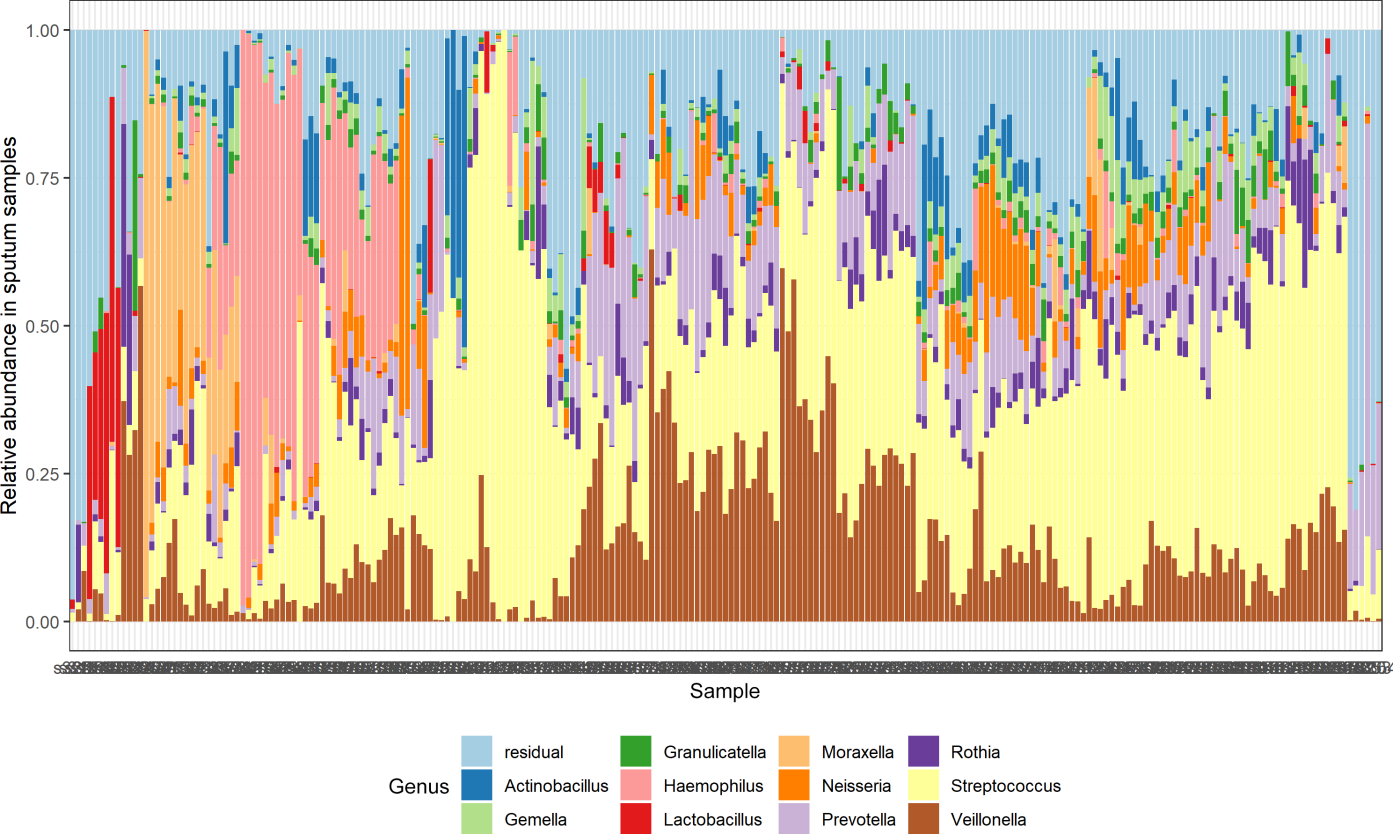
Distribution of the bacterial genera in sputum samples identified by 16S rRNA gene amplicon sequencing in 250 Xpert MTB/RIF-negative presumptive tuberculosis cases.

Haemophilus, *Streptococcus*, and *Moraxella* were among the most prevalent genera in the sputum samples of all study participants (Fig. 1). One or more potential bacterial LRTI pathogens were present at ≥1% in 79.1% (170/215) Xpert Ultra negative samples and 82.8% (29/35) Xpert Ultra positive samples (*P* = 0.615). In Xpert Ultra negative samples, *Haemophilus* spp. (*n* = 105, 48.7%), *Staphylococcus* spp. (*n* = 77, 35.8%), *S. pneumoniae*/*pseudopneumoniae* (*n* = 56, 26.0%), *Moraxella catarrhalis*/*nonliquefaciens* (*n* = 47, 21.9%), *M. pneumoniae* (*n* = 7, 3.3%), and *Bordetella pertussis* (*n* = 2, 0.9%) were detected on amplicon sequence variant (ASV) level. In addition, one or more opportunistic pathogens were identified in 40.8% of the 71 Xpert Ultra negative sputum samples collected from elderly patients or patients living with HIV: *Rothia aeria* (*n* = 24, 33.8%), *Acinetobacter baumannii* (*n* = 2, 2.8%), *Streptococcus pyogenes* (*n* = 1, 1.4%), and *P. aeruginosa* (*n* = 2, 2.8%). Except for *Mtb,* similar proportions of (potential) bacterial pathogens and opportunistic pathogens were detected in the Xpert Ultra positive sputum samples (Table 2). Multiple potential bacterial LRTI pathogens were more often identified in Xpert Ultra MTB-negative sputum samples (58.1%, 125 of 215) than in Xpert Ultra MTB-positive sputum samples (34.3%, 12 of 35) (*P* = 0.01).

**TABLE 2:** Potential bacterial pathogens identified by 16S rRNA gene amplicon sequencing in sputum samples stratified by Xpert MTB/RIF Ultra results

	Xpert Ultra negative	Xpert Ultra positive
All patients	215 (86.0)	35 (14.0)
Bacterial pathogens		
*Haemophilus* spp.	105 (48.7)	18 (51.4)
*Staphylococcus* spp.	77 (35.8)	12 (34.3)
*Streptococcus pneumoniae/pseudopneumoniae*	56 (26.0)	8 (22.9)
*Moraxella catarrhalis/nonliquefaciens*	47 (21.9)	9 (25.7)
*Mycoplasma pneumoniae*	7 (3.3)	0 (0.0)
*Mycobacterium tuberculosis*	0 (0.0)	5 (14.3)
*Bordetella pertussis*	2 (0.9)	0 (0.0)
HIV positive or elderly patients	71	12
Opportunistic pathogens		
*Rothia aeria*	24 (33.8)	3 (25.0)
*Pseudomonas aeruginosa*	2 (2.8)	0 (0.0)
*Acinetobacter baumannii*	2 (2.8)	0 (0.0)
*Streptococcus pyogenes*	1 (1.4)	1 (8.3)

### Association between bacterial pathogens in sputum sample and response to antibiotic trial

The sputum samples were collected from 215 patients with an Xpert Ultra negative result before they received a seven-day antibiotic trial of ceftriaxone and azithromycin (43.7%, *n* = 94), amoxicillin (30.2%, *n* = 65), or vancomycin plus doxycycline (26.1%, *n* = 56) (Fig. S1). Only 54.4% (*n* = 117) improved clinically after the antibiotic trial. Of 98 patients failing to respond to antibiotics, 21 started empiric TB treatment, whereas 77 did not. Of these 77 patients, the likely causative pathogen could be identified in the sputum sample of 7 (9%): *M. pneumoniae* (*n* = 2) *B. pertussis* (*n* = 2), *Acinetobacter baumanii* (*n* = 2), and *P. aeruginosa* (*n* = 1). In addition, 4 (5%) patients were diagnosed with bacteriologically confirmed TB during the 6-month follow-up period. For most 66 (86%) patients, the cause was their prolonged respiratory symptoms and their failure to respond to antibiotics remained unclear.

When adjusted for patient characteristics associated with poor response to an antibiotic trial (age ≥65 years, HIV status, history of TB treatment, presence of prolonged cough, fever, chest pain, or weight loss [Table S1]), the presence of *S. pneumoniae*/*pseudopneumoniae* (aOR 3.31, 95% CI 1.68–6.72), *Haemophilus* spp. (aOR 2.08, 95% CI 1.16–3.78), *M. catarrhalis*/*nonliquefaciens* (aOR 4.24, 95% CI 2.04–9.27), or *M. pneumoniae* (aOR 8.78, 95% CI 1.34–173.4) was associated with poor clinical response to an antibiotic trial (Table 3).

**TABLE 3:** Association between (potential) bacterial pathogens and response to antibiotic trial among 215 symptomatic hospitalized patients with an Xpert Ultra MTB/RIF-negative sputum result

Bacterial pathogens		Good response to antibiotic trial	Poor response to antibiotic trial	Crude OR (95% CI)	Adjusted OR^*a*^ (95% CI)
All patients		117 (54.4)	98 (45.6)		
*Haemophilus* spp.	Absent	70 (59.8)	40 (40.8)	ref	ref
	Present	47 (40.2)	58 (59.2)	**2.16 (1.25–3.75**)^*b*^	**2.08 (1.16–3.78**)
*Staphylococcus* spp.	Absent	81 (69.2)	57 (58.2)	ref	
	Present	36 (30.8)	41 (41.8)	1.62 (0.92–2.84)	
*Streptococcus pneumoniae/ pseudopneumoniae*	Absent	102 (87.2)	57 (58.2)	ref	ref
	Present	15 (12.8)	41 (41.8)	**4.89 (2.53–9.85**)	**3.31 (1.68–6.72**)
*Moraxella catarrhalis/nonliquefaciens*	Absent	102 (87.2)	66 (67.3)	ref	ref
	Present	15 (12.8)	32 (32.7)	**3.30 (1.68–6.70**)	**4.24 (2.04–9.27**)
*Mycoplasma pneumoniae*	Absent	116 (99.1)	92 (93.9)	ref	ref
	Present	1 (0.9)	6 (6.1)	**7.50 (1.26–144**)	**8.78 (1.34–173.4**)
*Bordetella pertussis*	Absent	117 (100)	96(98.0)	ref	
	Present	0 (0.0)	2 (2.0)	7.01 (4.8^e-64^-NA)	
Opportunistic bacterial pathogens					
HIV positive or elderly patients		31	40		
*Rothia aeria*	Absent	20 (64.5)	27 (57.4)	ref	
	Present	11 (35.5)	13 (32.5)	0.87 (0.32–2.37)	
*Pseudomonas aeruginosa*	Absent	30 (96.8)	39 (97.5)	ref	
	Present	1 (3.2)	1 (2.5)	0.77 (0.03–19.9)	
*Acinetobacter baumannii*	Absent	29 (93.5)	40 (100)	ref	
	Present	2 (6.5)	0 (0.0)	4.6e^−08^ (NA-1.9^e+108^)	
*Streptococcus pyogenes*	Absent	31 (100)	39 (97.5)	ref	
	Present	0 (0.0)	1 (2.5)	4.5e^+06^ (7.3^e-123^-NA)	

^*a*^ Adjusted for age ≥65 years, HIV status, history of TB treatment, presence of prolonged cough, fever, or chest pain, NA: infinitive number.

^*b*^ Boldface shows association.

### Association between bacterial pathogens in sputum sample and baseline chest X-ray findings

Overall, 29.2% of patients with an Xpert Ultra negative sputum sample had an abnormal chest X-ray. When adjusted for patient characteristics associated with the presence of an abnormal chest X-ray (rural residence, presence of prolonged cough, fever, chest pain, or shortness of breath [Table S2]), the odds of an abnormal chest X-ray were higher in the presence of *S. pneumoniae*/*pseudopneumoniae* (aOR 2.5, 95% CI 1.12–6.16) and lower in the presence of *M. catarrhalis*/*nonliquefaciens* (aOR 0.37, 95% CI 0.19–0.74) in the sputum (Table 4).

**TABLE 4:** Association between the presence of (potential) bacterial pathogens and chest X-ray findings among 215 symptomatic hospitalized patients with negative Xpert Ultra result

Bacterial pathogens		Chest X-ray findings^*b*^		Crude OR (95% CI)	Adjusted OR^*a*^ (95% CI)
		Normal	Abnormal		
All patients		**150** (**70.8**)^*c*^	**62** (**29.2**)		
*Haemophilus* spp.	Absent	81 (54.0)	26 (41.9)	ref	
	Present	69 (46.0)	36 (58.1)	1.62 (0.89–2.97)	
*Staphylococcus* spp.	Absent	95 (63.3)	41 (66.1)	ref	
	Present	55 (36.7)	21 (33.9)	0.88 (0.46–1.63)	
*Streptococcus pneumoniae/pseudopneumoniae*	Absent	117 (78.0)	40 (64.5)	ref	ref
	Present	33 (22.0)	22 (35.5)	1.95 (1.01–3.72)	**2.5 (1.12–6.16**)
*Moraxella catarrhalis/nonliquefaciens*	Absent	112 (74.7)	54 (87.1)	ref	ref
	Present	38 (25.3)	8 (12.9)	0.43 (0.17–0.95)	**0.37 (0.19–0.74**)
*Mycoplasma pneumoniae*	Absent	145 (96.7)	60 (96.8)	ref	
	Present	5 (3.3)	2 (3.2)	0.96 (0.13–4.62)	
*Bordetella pertussis*	Absent	149 (99.3)	61 (98.4)	ref	
	Present	1 (0.7)	1 (1.6)	2.44 (0.09–62.4)	
Opportunistic bacterial pathogens					
HIV positive or elderly patients		51	20		
*Rothia aeria*	Absent	33 (64.7)	14 (70.0)	ref	
	Present	18 (35.3)	6 (30.0)	0.78 (0.24–2.33)	
*Pseudomonas aeruginosa*	Absent	51 (100)	18 (90.0)	ref	
	Present	0 (0.00)	2 (10.0)	4.43 (1.1^e-108^- NA)	
*Acinetobacter baumannii*	Absent	50 (98.0)	19 (95.0)	ref	
	Present	1 (2.0)	1 (5.0)	2.63 (0.1–68.8)	
*Streptococcus pyogenes*	Absent	51 (100)	19 (95.0)	ref	
	Present	0 (0.00)	1 (5.0)	1.54 (2.5^e-122^ -NA)	

^*a*^ Adjusted for rural residence, presence of prolonged cough, fever, chest pain, or shortness of breath.

^*b*^ Three patients missing CXR diagnosis; NA: infinitive number.

^*c*^ Boldface shows association.

### Association between bacterial pathogens in sputum sample and survival status at 6 months

Among the 215 patients with a Xpert Ultra negative sputum, nine (4.2%) died: three while hospitalized and six after discharge. When adjusted for patient characteristics associated (*P* < 0.2) with survival status at 6 months (rural residence, body mass index, and HIV status [Table S3]), the presence of *Streptococcus pneumoniae*/*pseudopneumoniae* (aOR 5.31, 95% CI 1.29–26.6), *M. catarrhalis/nonliquefaciens* (aOR 12.1, 95% CI 2.67–72.8), and *M. pneumoniae* (aOR 34.5, 95% CI 4.79–292.3) were associated with mortality (Table 5). The presence of multiple pathogens was not associated with mortality among Xpert Ultra MTB-negative (OR 2.61, 95% CI 0.52–12.8) or Xpert Ultra MTB-positive patients (OR 2.10, 95% CI 0.25–17.1).

**TABLE 5:** Association between the presence of potential bacterial LRTI pathogens in sputum and mortality among 215 symptomatic hospitalized patients with negative Xpert Ultra result

Bacterial pathogens		Survival status		Crude OR (95% CI)	Adjusted OR^*a*^ (95% CI)
		Alive	Died		
*Haemophilus* spp.	Absent	107 (51.9)	3 (33.3)	ref	
	Present	99 (48.1)	6 (66.7)	2.1 (0.55–10.4)	
*Staphylococcus* spp.	Absent	132 (64.1)	6 (66.7)	ref	
	Present	74 (35.9)	3 (33.3)	0.8 (0.42–1.48)	
*Streptococcus pneumoniae*/ *pseudopneumoniae*	Absent	156 (75.7)	3 (33.3)	ref	ref
	Present	50 (24.3)	6 (66.7)	**6.2 (1.58–30.4**)^*b*^	**5.31 (1.29–26.6**)
*Moraxella catarrhalis*/*nonliquefaciens*	Absent	165 (80.1)	3 (33.3)	ref	ref
	Present	41 (19.9)	6 (66.7)	**8.0 (2.03–39.4**)	**12.1 (2.67–72.8**)
*Mycoplasma pneumoniae*	Absent	202 (98.1)	6 (66.7)	ref	ref
	Present	4 (1.9)	3 (33.3)	**25.2 (4.24–143**)	**34.5 (4.79–292.3**)
*Bordetella pertussis*	Absent	205(99.5)	8 (88.9)	ref	
	Present	1 (0.5)	1 (11.1)	25.6 (0.95–689)	
Opportunistic bacteria pathogens					
HIV positive or elderly patients		66	5		
*Rothia aeria*	Absent	44 (66.7)	3 (60.0)	ref	
	Present	22 (33.3)	2 (40.0)	1.33 (0.16–8.61)	
*Pseudomonas aeruginosa*	Absent	65 (98.5)	4 (80.0)	ref	
	Present	1 (1.5)	1 (20.0)	16.2 (0.57–468)	
*Acinetobacter baumannii*	Absent	64 (96.9)	5 (100)	ref	
	Present	2 (3.03)	0 (0.0)	3.38 (NA-3.3 ^e+183^)	
*Streptococcus pyogenes*	Absent	65 (98.5)	5 (100)	ref	
	Present	1 (1.5)	0 (0.0)	8.3 (NA- 1.2 ^e+206^)	

^*a*^ Adjusted for rural residence, body mass index, or HIV status. NA: Infinitive number.

^*b*^ Boldface shows association.

## DISCUSSION

In this study, we aimed to investigate the bacterial etiology of LRTI in patients presenting with symptoms of TB who had a very low probability of having TB given their sputum’s negative Xpert Ultra result based on 16S rRNA sequencing. The presence of potential bacterial pathogens in the sputum samples was identified and compared with their prevalence in Xpert Ultra positive sputum samples. We could determine the presence of most likely causal pathogen in only 13 of the 215 patients, as described in Table 2, with 7 cases of *M. pneumoniae*, 2 cases of *B. pertussis*, 2 cases of *A. baumannii*, and 2 cases of *P. aeruginosa*.

Overall, one or more (potential) bacterial LRTI pathogens were present in 80% of sputum Xpert Ultra negative samples. The most common pathogenic bacterial ASVs detected were *Haemophilus* spp.*, Staphylococcus* spp.*, Streptococcus pneumoniae (pseudo)pneumoniae,* and *M. catarrhalis*/*nonliquefaciens*, present in >20% of patients. The challenge in attributing LRTI to the presence of these pathogens is further highlighted by the observation that one or more of these (potential) bacterial LRTI pathogens were also present in about 82.8% of Xpert Ultra positive sputum samples and that, except for a higher prevalence of *Mtb* in Xpert Ultra positive sputum samples, the bacterial populations were almost similar for Xpert Ultra negative and positive samples.

This 80% prevalence of one or more potential bacterial LRTI pathogen is higher than what has been reported in Cameron and Cambodia based on culture methods, where bacterial LRTI pathogens was reported in 44% and 46.8% of presumptive TB cases, respectively (7, 25). The high prevalence of (potential) bacterial LRTI pathogens in patients with confirmed TB is in line with prior reports that co-detection with other bacterial pathogens is common in patients diagnosed with pulmonary TB (26, 27). In Cambodia, co-detection with another potential bacterial LRTI pathogens was observed in 33% of patients diagnosed with pulmonary TB by sputum culture (7). In Nigeria, 50% of sputum samples collected from patients with TB grew both *Mtb* and other bacteria implicated in LRTI as the same as in this paper (28). The higher prevalence may be explained by the use of 16S rRNA gene amplicon sequencing, which can identify both culturable and unculturable bacteria, providing a complete picture of the bacterial community of sputum samples (29, 30).

Among the patients with an Xpert Ultra negative sputum result, 29.2% had an abnormal chest X-ray, which is similar to the findings from a study in South Africa where 27.2% of Xpert Ultra negative patients had abnormal findings on chest X-ray (31). We also found that the presence of *Streptococcus pneumoniae*/*pseudopneumoniae* in the Xpert Ultra negative sputum samples increased the odds of an abnormal chest X-ray (aOR 2.5, 95% CI 1.12–6.16), whereas the presence of *M. catarrhalis*/*nonliquefaciens* decreased the odds of abnormal chest X-ray findings (aOR 0.37, 95% CI 0.19–0.74).

In our study population, just over half (54.4%) of patients with an Xpert Ultra negative sputum result improved on an antibiotic trial. Patients for whom *M. catarrhalis*/*nonliquefaciens, Streptococcus pneumoniae*/*pseudopneumoniae, M. pneumoniae,* and *Haemophilus* spp. was detected in the sputum sample had higher odds of poor response to an antibiotic trial, even after adjusting for patient characteristics. This may be due to the presence of drug-resistant bacteria (32). Three of the four pathogens associated with failure to improve on an antibiotic trial were also associated with an increased odds of mortality in the 6 months following the initial assessment: *S. pneumoniae/pseudopneumoniae* (aOR 5.31, 95% CI 1.29–26.6), *M. catarrhalis/nonliquefaciens* (aOR 12.1, 95% CI 2.67–72.8), and *M. pneumoniae* (aOR 34.5 95% CI 4.79–292.3).

The main strength of the study was the use of 16S rRNA sequencing for the first time to detect bacterial LRTI pathogens in sputum samples of patients presenting with symptoms of TB who had a very low probability of having TB as *Mtb* was not detected by the highly sensitive Xpert Ultra assay. Another strength is the prospective collection of comprehensive clinical data. This allowed an assessment of the associations between the bacterial community and patient outcomes. Our study also had some limitations. First, this was a hospital-based study, limiting generalizability to outpatient settings. Second, bacterial sputum culture was not available in our resource-poor study setting, and assessment of sputum quality using Gram staining to determine the extent of oral flora contamination was not performed. A positive result from 16S rRNA gene sequencing may indicate either infection or colonization of the normal respiratory flora (29). Third, despite using the DADA2 algorithm with ASVs to increase the sensitivity and specificity compared to OUT picking methods, the 16S rRNA amplicon sequencing of the V4 region could not always discriminate accurately up to species level. For instance, of the *Haemophilus* spp.*, Haemophilus influenza* type b and non-typable *Haemophilus* are causal pathogens for LRTI (33). However, *Haemophilus parainfluenza* is a common isolate from the healthy nasopharynx as well as *H. influenzae* type b. Non-typable *H. influenzae* can be found in sputum cultures of nearly half of adults with chronic bronchitis (34). Finally, because it is unclear which level of abundance a pathogen is clinically relevant, we reported any presence above 1%. This may have resulted in the inclusion of minority populations of pathogenic bacteria that are not of clinically important.

Among the overall 30 types of the *Staphylococcus* spp.*, S. aureus* is a common cause of pneumonia, but it is also frequently isolated in respiratory samples from healthy individuals as a colonizing bacterium (35). Of the *Streptococcus* spp.*, S. pneumoniae* is a well-established cause of LRTI, but the role of *S. (pseudo)pneumoniae* is less certain, although it has been reported in COPD (36). *M. nonliquefaciens* frequently colonizes the upper respiratory tract and is usually non-pathogenic, rarely causing invasive disease (37). *M. catarrhalis* also commonly colonizes the healthy airways (38), but it can cause pneumonia in children and adults with underlying chronic lung disease (39). Third, although viral and fungal communities can cause LRTI, they cannot be detected in sputum samples when using 16 s rRNA. Finally, as drug susceptibility tests were not performed, the presence of antibiotic resistance as a cause for poor response to an antibiotic trial or mortality could not be assessed.

In conclusion, the study found that 16S rRNA could identify the bacterial pathogen responsible for LRTI in 6.0% of Xpert Ultra negative patients but was not specific enough to differentiate between carriage and disease-causing pathogens in 80% of cases, making this approach not appear to be clinically useful. The presence of *M. pneumonia* was associated with 34 times greater odds of mortality and the presence of *S. pneumoniae* (*pseudo*)*pneumoniae* or *M. catarrhalis*/*nonliquefaciens* increased the odds of mortality rate by 5 to 12 times, respectively, suggesting clinical relevance of these pathogens. Further research using tools with higher discriminatory power that can also detect viruses and fungi is required to guide the management of Xpert Ultra negative patients.

## MATERIALS AND METHODS

### Study site, design, and data collection

We performed a secondary analysis of a cohort study that aimed to determine the impact of empiric TB treatment on mortality among hospitalized patients who tested negative on the Xpert MTB/RIF assay (13). In this cohort study, sputum samples were collected before antibiotic trials were started from 250 adults (age  ≥18 years) with symptoms of pulmonary TB (current cough, night sweats, fever, and weight loss) who were hospitalized between December 2018 to July 2019 in the Jimma Medical Center in Ethiopia. At the Jimma University Mycobacteriology Research Center, the TB reference laboratory for Southwest Ethiopia, sputum samples were decontaminated and evaluated for the presence of *Mtb* by liquid culture using the Mycobacteria Growth Indicator Tube (MGIT) BACTEC MGIT 960 System (Becton Dickinson, Sparks, MD, USA), solid Lowenstein-Jensen (LJ) media culture, and the Xpert Ultra assay (Cepheid, Sunnyvale, CA, USA) (40, 41). The Xpert Ultra test was repeated on the same sample in case of an invalid result and repeated on another sample in case of a “trace” result. Ethical clearance was obtained from the Ethical Review Board of Institute of Health, Jimma University, with Ref. No: IHRPGD/397/2018. Written informed consent was obtained from all study participants. A structured questionnaire was used to collect demographic and clinical data; medical records were reviewed for HIV status, chest X-ray findings, and response to antibiotic treatment. All study participants were followed up for 6 months to determine survival status.

### 16S rRNA gene amplicon sequencing

DNA was extracted from stored unprocessed sputum samples (stored at −80°C for 24 months) at the Mycobacteriology Research Center of Jimma University in Ethiopia using the commercially available PowerFecal DNA Isolation Kit (Qiagen) (42). MiSeq preparations were done in the Lab of Applied Microbiology and Biotechnology (Belgium) using an in-house optimized protocol for low-biomass samples (38), and dual-index paired-end Illumina MiSeq 16S rRNA V4 region with an amplicon size of 254-bp sequencing was performed at the Center for Medical Genetics of the University of Antwerp (Belgium), as described (38).

### Statistical analysis

Processing and quality control of the sequencing reads were performed using the R package Divisive Amplicon Denoising Algorithm 2 (DADA2), version 1.6.0., to increase the sensitivity and specificity compared to OUT picking methods (38). At the genus level, we processed amplicon sequence variants (ASVs) and aggregated ASV read counts. We annotated ASVs and added metadata to samples using R. Statistical analyses and data visualization was performed using R.

Bacteria were categorized as present in the sputum sample when they were present at ≥1% of the population. Bacteria were then classified as potentially pathogenic, opportunistic (i.e., cause of disease in immunocompromised individuals, including people living with HIV or elderly people), or not LRTI-causing based on literature review using PubMed, ScienceDirect, and Google Scholar and using search terms pathogenic bacteria, opportunistic bacteria, bacterial genera, LRTI, and bacterial classification (Table S4). When comparison of the 16S rRNA amplicon data could not classify the bacteria present to species level, the bacteria present were classified as potential LRTI pathogens.

The difference of bacterial LRTI pathogens detected was compared between Xpert Ultra positive and negative samples using chi-squared test. Logistic regression analysis was performed to determine the association (odds ratio [OR] and its 95% CI) between (potential) bacterial LRTI pathogens and response to an antibiotic trial, findings on chest X-ray, and 6-month survival status. For each (potential) bacterial LRTI pathogen identified and each outcome of interest, a separate model was built. For each model, the adjusted OR was estimated by including patient characteristics that were associated with the outcome of interest at *P*-value < 0.2 in bivariate analysis. Generalized variance-inflation factor was estimated to check multicollinearity. Backward stepwise model reduction was performed using the likelihood ratio test with a *P*-value cut-point of 0.1.

## Data Availability

The sequence data used and/or analyzed during the current study are included as supplemental material.

## SUPPLEMENTAL MATERIAL

Data Set S1 spectrum.02931-23-s0001.csv Sequence data.

Supplemental material spectrum.02931-23-s0002.docx Additional experimental details, figures, and tables.

ASM does not own the copyrights to Supplemental Material that may be linked to, or accessed through, an article. The authors have granted ASM a non-exclusive, world-wide license to publish the Supplemental Material files. Please contact the corresponding author directly for reuse.
