# Supplementary material for: Bacterial pathogens in Xpert MTB/RIF Ultra-negative sputum samples of patients with presumptive tuberculosis in a high TB burden setting: a 16S rRNA analysis
Source: Microbiol Spectr. 2024 Jan 8;12(2):e02931-23. doi: 10.1128/spectrum.02931-23 (PMC10845949; doi:10.1128/spectrum.02931-23)
Supplement: Supplemental material — Additional experimental details, figures, and tables. [file spectrum.02931-23-s0002.docx]

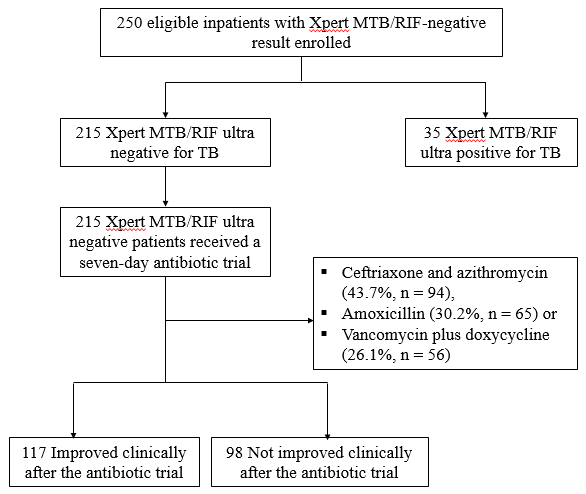


**Fig. S1:** Flow chart of enrollment, result of TB diagnosis, and antibiotic trial among 250 Xpert-negative adults hospitalized at Jimma University Medical Center, Ethiopia.

**Table S1**: Association between patient characteristics and response to an antibiotic trial.

| **Characteristics** | **Category** | **Clinical improvement on antibiotic trial** | **No clinical improvement on antibiotic trial** | **Crude OR**  **(95% CI)** | **P value** |
| --- | --- | --- | --- | --- | --- |
| Age (in years) | 18- 40 | 66 (56.4) | 47 (47.9) | ref |  |
|  | 41-64 | 38 (32.4) | 35 (35.7) | 1.2 (0.71-2.34) | 0.394 |
|  | ≥65 | 13 (11.2) | 16 (16.4) | 1.7 (0.76-3.98) | 0.192 |
| Sex | Female | 65 (55.5) | 52 (53.1) | ref |  |
|  | Male | 52 (44.5) | 46 (46.9) | 1.1 (0.64-1.89) | 0.715 |
| Residence | Urban | 50 (42.7) | 42 (42.8) | ref |  |
|  | Rural | 67 (57.3) | 56 (57.2) | 0.9 (0.57-1.71) | 0.986 |
| Body mass index | <18.5 | 58 (49.5) | 41 (41.8) | ref |  |
|  | 18.5-24.9 | 31(26.5) | 30 (30.6) | 1.3 (0.72-2.60) | 0.337 |
|  | >25-29.9 | 28 (24.0) | 27 (27.6) | 1.3 (0.70-2.65) | 0.358 |
| HIV status* | Negative | 96 (83.5) | 70 (75.2) | ref |  |
|  | Positive | 19 (16.5) | 23 (24.7) | 1.6 (0.84-3.30) | 0.144 |
| History of TB treatment | No | 91 (77.8) | 87 (88.8) | ref |  |
|  | Yes | 26 (22.2) | 11 (11.2) | 0.4 (0.19-0.92) | 0.036 |
| Weight loss | No | 47 (40.2) | 56 (57.1) | ref |  |
|  | Yes | 70 (59.8) | 42 (42.9) | 0.5 (0.29-0.86) | 0.0136 |
| Cough | <2 weeks | 39 (33.3) | 42 (42.8) | ref |  |
|  | ≥2weeks | 78 (66.7) | 56 (57.2) | 0.6 (0.38-1.16) | 0.152 |
| Shortness of breath | <2 weeks | 58 (49.6) | 56 (57.2) | ref |  |
|  | ≥ 2 weeks | 59 (50.4) | 42 (42.8) | 0.7 (0.42-1.26) | 0.268 |
| Night sweat | <2 weeks | 56 (47.9) | 57 (58.1) | ref |  |
|  | ≥ 2 weeks | 61 (52.1) | 41 (41.8) | 0.6 (0.38-1.13) | 0.133 |
| Fever | <2 weeks | 62 (53.0) | 59 (60.2) | ref |  |
|  | ≥ 2 weeks | 55 (47.0) | 39 (39.8) | 0.7(0.43-1.28) | 0.289 |
| Loss of appetite | <2 weeks | 27 (23.1) | 20 (20.4) | ref |  |
|  | ≥ 2 weeks | 90 (76.9) | 78 (79.6) | 1.1 (0.61-2.26) | 0.637 |
| Chest pain | <2 weeks | 35 (29.9) | 43 (43.9) | ref |  |
|  | ≥ 2 weeks | 82 (70.1) | 55 (56.1) | 0.5 (0.30-0.95) | 0.034 |

* HIV status missing for 7 Xpert Ultra negative patients

**Table S2**: Association between patient characteristics and chest X-ray findings among 215 symptomatic hospitalized patients with negative Xpert Ultra result.

| **Characteristics** | **Category** | **Chest X-ray findings** | | **Crude OR (95% CI)** | P value |
| --- | --- | --- | --- | --- | --- |
|  |  | **Normal** | **Abnormal** |  |  |
| Age (in years) | 18- 40 | 81 (54.0) | 29 (46.8) | ref |  |
|  | 41-64 | 49 (32.7) | 24 (38.7) | 1.36 (0.71-2.61 | 0.342 |
|  | ≥65 | 20 (13.3) | 9 (14.5) | 1.25 (0.40-3.01) | 0.616 |
| Sex | Female | 81 (54.0) | 35 (56.5) | ref |  |
|  | Male | 69 (46.0) | 27 (43.5) | 0.9 (0.5-1.64) | 0.744 |
| Residence | Urban | 68 (45.3) | 22 (35.5) | ref |  |
|  | Rural | 82 (54.7) | 40 (64.5) | 1.5 (0.82-2.81) | 0.188 |
| Body mass index | <18.5 | 72 (48.0) | 25 (40.3) | ref |  |
|  | 18.5-24.9 | 43 (28.7) | 18 (29.0) | 1.20 (0.58-2.45) | 0.608 |
|  | >25-29.9 | 35 (23.3) | 19 (30.7) | 1.56 (0.75-3.21) | 0.224 |
| HIV status* | Negative | 114 (79.2) | 49 (80.3) | ref |  |
|  | Positive | 30 (20.8) | 12 (19.7) | 0.93 (0.42-1.93) | 0.851 |
| History of TB treatment | No | 124 (82.7) | 51 (82.3) | ref |  |
|  | Yes | 26 (17.3) | 11 (17.7) | 1.02 (0.45-2.19) | 0.943 |
| Weight loss | No | 84 (56.0) | 19 (30.6) | ref |  |
|  | Yes | 66 (44.0) | 43 (69.4) | 2.88 (1.55-5.49) | 0.0001 |
| Cough | <2 weeks | 64 (42.7) | 17 (27.4) | ref |  |
|  | ≥2weeks | 86 (57.3) | 45 (72.6) | 1.96 (1.04-3.83) | 0.0394 |
| Shortness of breath | <2 weeks | 86 (57.3) | 27 (43.5) | ref |  |
|  | ≥ 2 weeks | 64 (42.7) | 35 (56.5) | 1.74 (0.96-3.18) | 0.0686 |
| Night sweat | <2 weeks | 80 (53.3) | 31 (50.0) | ref |  |
|  | ≥ 2 weeks | 70 (46.7) | 31 (50.0) | 1.14 (0.63-2.07) | 0.659 |
| Fever | <2 weeks | 90 (60.0) | 29 (46.8) | ref |  |
|  | ≥ 2 weeks | 60 (40.0) | 33 (53.2) | 1.7 (0.94-3.11) | 0.0788 |
| Loss of appetite | <2 weeks | 33 (22.0) | 13 (21.0) | ref |  |
|  | ≥ 2 weeks | 117 (78.0) | 49 (79.0) | 1.06 (0.52-2.25) | 0.8687 |
| Chest pain | <2 weeks | 69 (46.0) | 9 (14.5) | ref |  |
|  | ≥ 2 weeks | 81 (54.0) | 53 (85.5) | 5.01 (2.4-11.5) | 4.65e-05 |

* HIV status is missing for six patients with normal chest X-ray findings and one with abnormal chest X-ray findings.

**Table S3:** Association between patient characteristics and six-month mortality among 215 symptomatic hospitalized patients with negative Xpert Ultra result.

| **Characteristics** | **Category** | **Survival status** | | **Crude OR**  **(95% CI)** | **P value** |
| --- | --- | --- | --- | --- | --- |
|  |  | **Alive** | **Died** |  |  |
| Age (in years) | 18- 40 | 109 (52.9) | 4 (44.4) | ref |  |
|  | 41-64 | 69 (33.5) | 4 (44.4) | 1.5 (0.36-6.87) | 0.527 |
|  | ≥65 | 28 (13.6) | 1 (11.2) | 0.9 (0.04-6.90) | 0.981 |
| Sex | Female | 113 (54.9) | 4 (44.4) | ref |  |
|  | Male | 93 (45.1) | 5 (55.6) | 1.5 (0.39-6.28) | 0.542 |
| Residence | Urban | 86 (41.7) | 6 (66.7) | ref |  |
|  | Rural | 120 (58.3) | 3 (33.3) | 0.3 (0.07-1.39) | 0.155 |
| Body mass index (kg·m^−2^) | <18.5 | 97 (47.1) | 2 (22.3) | ref |  |
|  | 18.5-24.9 | 58 (28.1) | 3 (33.3) | 2.5 (0.40-19.4) | 0.322 |
|  | >25-29.9 | 51 (24.8) | 4 (44.4) | 3.8 (0.03-1.39) | 0.130 |
| HIV status* | Negative | 161 (80.9) | 5 (55.6) | ref |  |
|  | Positive | 38 (19.1) | 4 (44.4) | 3.3 (0.8-13.4) | 0.078 |
| History of TB treatment | No | 171 (83.0) | 7 (77.7) | ref |  |
|  | Yes | 35 (17.0) | 2 (22.3) | 1.4 (0.20-6.06) | 0.685 |
| Weight loss | No | 100 (48.5) | 3 (33.3) | ref |  |
|  | Yes | 106 (51.5) | 6 (66.7) | 1.88 (0.48-9.12) | 0.378 |
| Cough | <2 weeks | 78 (37.8) | 3 (33.3) | ref |  |
|  | ≥2weeks | 128 (62.2) | 6 (66.7) | 1.2 (0.31-5.90) | 0.784 |
| Shortness of breath | <2 weeks | 109 (52.9) | 5 (55.6) | ref |  |
|  | ≥ 2 weeks | 97 (47.1) | 4 (44.4) | 0.8 (0.21-3.48) | 0.876 |
| Night sweat | <2 weeks | 107 (51.9) | 6 (66.7) | ref |  |
|  | ≥ 2 weeks | 99 (48.1) | 3 (33.3) | 0.5 (0.11-2.10) | 0.393 |
| Fever | <2 weeks | 116 (56.3) | 5 (55.6) | ref |  |
|  | ≥ 2 weeks | 90 (43.7) | 4 (44.4) | 1.0 (0.24-4.0) | 0.964 |
| Loss of appetite | <2 weeks | 46 (22.3) | 1 (11.2) | ref |  |
|  | ≥ 2 weeks | 160 (77.7) | 8 (88.8) | 2.3 (0.40-43.2) | 0.437 |
| Chest pain | <2 weeks | 75 (36.4) | 3 (33.3) | ref |  |
|  | ≥ 2 weeks | 131 (63.6) | 6 (66.7) | 1.1 (0.29-5.54) | 0.851 |
| Empiric TB-treatment | No | 117 (56.8) | 4 (44.4) | ref |  |
|  | Yes | 89 (43.2) | 5 (55.6) | 1.6 (0.42- 6.80) | 0.469 |

**Table S4:** Classification of bacteria as pathogenic or opportunistic (i.e., causative of LRTI in people living with HIV or the elderly) when detected in clinical sputum specimens of patients with clinical suspicion of LRTIs.

| **Authors** | **Year of publication** | **Type of samples used** | **Methods** | **Identified bacterial pathogens** | **Opportunistic bacteria** | **Ref.** |
| --- | --- | --- | --- | --- | --- | --- |
| Zacharioudakis IM, et al. | 2021 | Sputum | BioFire pneumonia pannel | *Streptococcus pneumonia, Staphylococcus Spp., Klebsiella pneumoniae, Haemophilus influenzae, Moraxella catarrhalis, Klebsella aerogenes, Mycoplasma spp.* | *Rothia aeria Streptococcus pyrogens, Streptococcus constellatus*, *Acinetobacter baumannii, Pseudomonas aeruginosa* | [[1](#_ENREF_1)] |
| Buchan BW, et. | 2022 | Sputum, Broncho alveolar lavage tracheal aspirate) | BioFire Film Array pneumonia panel and  Unyvero LRT, BAL panel | *Streptococcus pneumonia, Staphylococcus Spp., Legionella pneumophila, Klebseilla pneumoniae, Haemophilus influenzae, Moraxella catarrhalis, Klebsella aerogenes, Mycoplasma spp.* | *Streptococcus pyrogens*, *Pseudomonas aeruginosa, Acinetobacter baumannii* | [[2](#_ENREF_2)] |
| Alby K, et al | 2018 | Sputum and endotracheal aspirates | BioFire Film Array pneumonia panel | *Pseudomonas aeruginosa, Klebseilla pneumoniae, Haemophilus influenzae, Moraxella catarrhalis, Proteus spp.* | *Streptococcus pyrogens, Streptococcus constellatus*, *Acinetobacter baumannii* | [[3](#_ENREF_3)] |
| Kamel NA, et al | 2022 | Broncho alveolar lavage | FilmArray Pneumonia Panel plus | *Streptococcus pneumonia, Staphylococcus Spp., Klebseilla pneumoniae, Haemophilus influenzae, Escherichia coli, Moraxella catarrhalis, Serratia marcecens* | *Acinetobacter baumannii, Pseudomonas aeruginosa* | [[4](#_ENREF_4)] |
| Yoo IY, et al | 2020 | Sputum | FilmArray Pneumonia Panel plus | *Streptococcus pneumonia, Staphylococcus Spp., Klebseilla pneumoniae, Haemophilus influenzae, Escherichia coli, Klebsella aerogenes, Klebsiella oxytoca.* | *Acinetobacter baumannii,* *Pseudomonas aeruginosa* | [[5](#_ENREF_5)] |
| Xie G, et al | 2021 | Sputum, bronchoalveolar lavage fluid, lung tissue by transbronchial lung biopsy, pleural effusion, and blood | Metagenomic next-generation sequencing | *Streptococcus pneumonia, Streptococcus pseudopneumonia, Staphylococcus Spp., Entrococcus faecium, Pseudomonas aeruginosa, Klebseilla pneumoniae, Haemophilus influenzae, Acinetobacter baumannii, Moraxella catarrhalis, Klebsella aerogenes.* | *Rothia aeria, Streptococcus pyrogens, Acinetobacter baumannii* | [[6](#_ENREF_6), [7](#_ENREF_7)] |
| Huang J, et al. | 2020 | Sputum and bronchoalveolar lavage fluid | Metagenomic next-generation sequencing | *Streptococcus pneumonia, Haemophilus influenzae, Streptococcus pseudopneumonia,* | *Rothia aeria,* *Acinetobacter baumannii*, *Pseudomonas aeruginosa* | [[8](#_ENREF_8)] |

**References**

1. Zacharioudakis IM, Zervou FN, Dubrovskaya Y, Inglima K, See B, Aguero-Rosenfeld M. Evaluation of a Multiplex PCR Panel for the Microbiological Diagnosis of Pneumonia in Hospitalized Patients: Experience from an Academic Medical Center. International journal of infectious diseases : IJID : official publication of the International Society for Infectious Diseases. 2021;104:354-60. Epub 2021/01/13. doi: 10.1016/j.ijid.2021.01.004. PubMed PMID: 33434669.
2. Buchan BW, Windham S, Balada-Llasat JM, Leber A, Harrington A, Relich R, et al. Practical Comparison of the BioFire FilmArray Pneumonia Panel to Routine Diagnostic Methods and Potential Impact on Antimicrobial Stewardship in Adult Hospitalized Patients with Lower Respiratory Tract Infections. Journal of clinical microbiology. 2020;58(7). Epub 2020/05/01. doi: 10.1128/jcm.00135-20. PubMed PMID: 32350045; PubMed Central PMCID: PMCPMC7315039.
3. Alby K, Mitchell SL. Lower Respiratory Multiplex Panels for the Detection of Bacterial and Viral Infections. Clinical microbiology newsletter. 2018;40(16):131-6. Epub 2018/08/15. doi: 10.1016/j.clinmicnews.2018.07.005. PubMed PMID: 32287689; PubMed Central PMCID: PMCPMC7132739.
4. Kamel NA, Alshahrani MY, Aboshanab KM, El Borhamy MI. Evaluation of the BioFire FilmArray Pneumonia Panel Plus to the Conventional Diagnostic Methods in Determining the Microbiological Etiology of Hospital-Acquired Pneumonia. Biology. 2022;11(3). Epub 2022/03/27. doi: 10.3390/biology11030377. PubMed PMID: 35336751; PubMed Central PMCID: PMCPMC8945136.
5. Yoo IY, Huh K, Shim HJ, Yun SA, Chung YN, Kang OK, et al. Evaluation of the BioFire FilmArray Pneumonia Panel for rapid detection of respiratory bacterial pathogens and antibiotic resistance genes in sputum and endotracheal aspirate specimens. International journal of infectious diseases : IJID : official publication of the International Society for Infectious Diseases. 2020;95:326-31. Epub 2020/03/18. doi: 10.1016/j.ijid.2020.03.024. PubMed PMID: 32179139.
6. Xie G, Zhao B, Wang X, Bao L, Xu Y, Ren X, et al. Exploring the Clinical Utility of Metagenomic Next-Generation Sequencing in the Diagnosis of Pulmonary Infection. Infectious diseases and therapy. 2021;10(3):1419-35. Epub 2021/06/13. doi: 10.1007/s40121-021-00476-w. PubMed PMID: 34117999; PubMed Central PMCID: PMCPMC8322361.
7. Sonehara K, Araki T, Hanaoka M. Rothia aeria pneumonia in an immunocompetent patient: A novel case study. Respirology case reports. 2021;9(10):e0843. Epub 2021/09/30. doi: 10.1002/rcr2.843. PubMed PMID: 34584726; PubMed Central PMCID: PMCPMC8455964.
8. Huang J, Jiang E, Yang D, Wei J, Zhao M, Feng J, et al. Metagenomic Next-Generation Sequencing versus Traditional Pathogen Detection in the Diagnosis of Peripheral Pulmonary Infectious Lesions. Infection and drug resistance. 2020;13:567-76. Epub 2020/02/29. doi: 10.2147/idr.s235182. PubMed PMID: 32110067; PubMed Central PMCID: PMCPMC7036976.
